# Use of esketamine for tracheoscopic drug injection: a randomized controlled trial

**DOI:** 10.3389/fmed.2024.1479741

**Published:** 2024-10-25

**Authors:** Xiaoming Zhao, Zhendong Zhou, Zhenhua Li, Zhaolan Hu, Yuanyuan Yu

**Affiliations:** Department of Anesthesia, Qingdao Traditional Chinese Medicine Hospital (Qingdao Hiser Hospital), Qingdao Hiser Hospital Affiliated of Qingdao University, Qingdao, China

**Keywords:** esketamine, sufentanil, tracheoscopy, curative effect, security

## Abstract

**Background:**

Sufentanil may induce hypotensive bradycardia and other adverse reactions in elderly patients during anesthesia, while esketamine exhibits sedative and analgesic effects with minimal impact on respiration and circulation. The objective of this study was to investigate the impact of these two anesthetics on vital signs in patients undergoing bronchoscopy and lavage under general anesthesia.

**Method:**

This study was a randomized controlled trial with a parallel design. A total of 100 patients aged ≥60 years with ASAI or II who were undergoing bronchoscopy and lavage were randomly assigned to two groups: group A (esketamine, *n* = 50) and group B (sufentanil, *n* = 50). During anesthesia induction, both groups received intravenous infusion of propofol at a dose of 1.5 mL/kg and atracurium. In group A, esketamine at a dose of 0.3 mg/kg was injected; in group B, sufentanil at a dose of 0.2 μg/kg was injected intravenously. Subsequently, a laryngeal mask was inserted and connected to an anesthesia machine for mechanical ventilation. Anesthesia maintenance involved continuous intravenous infusion of propofol at a dose of 3 mL/kg. The mean arterial pressure (MAP), heart rate (HR), and blood oxygen saturation (SpO2) were recorded at various time points: before anesthesia injection (T0), after laryngeal mask insertion (T1), 5 min after the operation started (T2), 15 min after the operation started (T3), and before the end of the operation (T4). Additionally, the recovery time was recorded.

**Results:**

The blood pressure of patients in the esketamine group exhibited higher levels compared to those in the sufentanil group at multiple time points during the operation, while maintaining a more stable intraoperative blood pressure and shorter postoperative recovery time than that observed in the sufentanil group. The blood pressure and heart rate of patients in the esketamine group exhibited significant fluctuations after laryngeal mask implantation compared to pre-anesthesia induction, with a statistically significant increase observed. Conversely, no significant changes were observed in the sufentanil group. The heart rate and oxygen saturation showed no significant differences between the two groups, nor did the amount of propofol administered during the procedure.

**Conclusion:**

The utilization of esketamine during the induction phase of bronchoscopy and lavage under general anesthesia can enhance hemodynamic stability and reduce the occurrence of hypotension, thereby facilitating postoperative anesthetic recovery.

## 1 Introduction

Malignant tumor cells invade the trachea, leading to malignant tracheal stenosis, which can cause dyspnea and is considered a severe respiratory disease. Therefore, the treatment primarily aims to immediately eliminate airway obstruction. Previous studies have shown that the intratumoral injection of cisplatin and Endostar, especially when combined with cryotherapy and hyperthermia, under a bronchoscope can eliminate tumors, control tumor growth, maintain a smooth trachea and bronchial lumen in a timely manner, and prolong the time of tracheal restenosis (1, 2). However, it can also lead to several issues. Patients can feel uncomfortable during the treatment, particularly in the long-term treatment process, commonly patients cannot be treated or treatment fails because they cannot cooperate. To improve the comfort and efficacy of examination and treatment, general anesthesia is preferred. In this anesthetic method, sufentanil is commonly used in the induction stage. However, the use of sufentanil in elderly patients can often cause adverse events such as cough, chest wall stiffness, hypotension, and bradycardia (3), which can affect surgeries using a tracheoscope. Esketamine is the dextral structure of ketamine, which has effects including sedation, analgesia, and loss of consciousness. However, it has a minimal effect on respiration and circulation (4). Therefore, this study evaluated the efficacy and safety of ketamine combined with propofol for painless tracheoscopy in patients with tumors to provide a reference for clinical medication.

## 2 Methods

### 2.1 Clinical data

We collected the data of 100 patients who underwent painless bronchoscopy and received injection therapy from January 2022 to March 2023 at the Department of Respiratory and Critical Care Medicine, Qingdao Haici Hospital Affiliated to Qingdao University. All patients presented with The American Society of Anesthesiologists (ASA) classification I–II disease. However, none of the patients were at high risk for anesthesia. Inclusion criteria: The inclusion criteria were as follows: patients with lung cancer, lung metastases, and tracheal tumors confirmed on pathology and/or cytology who had central airway lesions that were intratracheal + wall or wall type, those who required painless bronchoscopy and injection therapy under a bronchoscope, and those aged over 60 years. Exclusion criteria: The exclusion criteria were as follows: patients allergic to narcotic drugs; those who refused to undergo tracheoscopy; those with uncontrolled hypertension, cerebral infarction within the last 3 months, oral anticoagulants, new cardiovascular events, and oral antiplatelet drugs that cannot be discontinued; those with severe respiratory insufficiency or cardiac insufficiency who could not tolerate tracheoscopy and treatment; and those with vital signs that changed due to excessive bleeding during surgery. The participants were randomly divided into group A who included patients receiving esketamine (*n* = 50) and group B who comprised patients receiving sufentanil (*n* = 50).

### 2.2 Anesthetic methods

During anesthesia induction, propofol at a dose of 1.5 mL/kg and atracurium at a dose of 0.4 mg/kg were intravenously administered. Group A received esketamine at a dose of 0.3 mg/kg intravenously. Group B received sufentanil at a dose of 0.2 μg/kg intravenously. A laryngeal mask was inserted and connected to the anesthesia machine for mechanical ventilation. The tidal volume was calculated based on the standard of 8–10 mL/kg, while end-tidal carbon dioxide levels were maintained within the range of 35–45 mmHg. Anesthesia was maintained via continuous intravenous infusion with propofol at a dose of 3 mg/kg.

### 2.3 Surgical method using the tracheoscope

The patients were treated with a bronchoscope injection needle (Shanghai Elton Model: af-d1810pn). Routine bronchoscopy was performed. After cutting the tumor under a bronchoscope, the needle was inserted into the residual tumor under direct visualization. Injection was performed at 4–6 points at the center and periphery of the tumor. The penetration depth was 3–4 mm. Endostar (15 mg) and cisplatin (20 mg) were diluted to 4 mL. Four to six parts of the tumor were injected alternately with 0.5 mL of drugs each time, twice a week for two consecutive weeks.

### 2.4 Observation index

The primary outcomes, including mean arterial pressure (MAP), heart rate (HR), and blood oxygen saturation, were recorded at multiple time points: before anesthesia injection (T0), after laryngeal mask insertion (T1), 5 min after the beginning of surgery (T2), 15 min after the beginning of surgery (T3), and before the end of surgery (T4). The secondary outcome, the time from the end of the procedure until the patient can open his eyes and cooperate with commands.

### 2.5 Quality control plan

All examinations were performed by the same physician and assistant and by the same anesthesiologist.

### 2.6 Sample size estimation

According to the results of the preliminary experiment, the mean arterial pressure (MAP) in the sufentanil group was 96 ± 10 mmHg after laryngeal mask airway (LMA) insertion. The predicted increase in MAP in the esketamine group was 7 mmHg (mean MAP value of 103 ± 10 mmHg), with a two-sided *α* = 0.05 and a power of 90%. The sample size calculation using PASS 15 software yielded a requirement of 88 subjects. Considering a potential dropout rate of 20%, at least 100 subjects were ultimately needed.

### 2.7 Statistical processing

SPSS 23.0 software was utilized for conducting statistical analysis. The Shapiro–Wilk test was employed to assess the normality of the data. The qualitative data are illustrated in the form of examples (%) and compared using chi-square or Fisher exact tests. Quantitative data were expressed as mean ± SD, with group comparisons conducted using t-tests and comparisons at different time points performed through repeated measures analysis of variance (ANOVA). The within-group comparisons were conducted using a multivariate analysis of variance. Statistical significance was defined as a *p* value less than 0.05.

## 3 Results

### 3.1 Demographic data and clinical characteristics

There were no significant differences in terms of age, sex, body mass index, and ASA classification (I/II) between the two groups (*p* > 0.05), as shown in Table 1.

**Table 1:** Preoperative demographics and characteristics.

Baseline indicators	Group A	Group B	*p*
Sex (M/F)	32 (64)/18 (36)	29 (58)/21 (42)	0.681
Age (years)	66.9 ± 4.8	68.1 ± 6.5	0.405
BMI (kg/m^2^)	23.2 ± 2.3	23.6 ± 2.7	0.427
ASA I/II	19 (38)/31 (62)	22 (44)/28 (56)	0.068

BMI, body mass index; ASA, American Society of Anesthesiologists physical status. Sex and ASA were expressed as number of patients (%), Age and BMI as mean ± SD. The significance level of *p* < 0.05 indicates a statistically significant difference between the two groups.

### 3.2 Recovery time for anesthesia

The recovery time from anesthesia in group A was lower than that in group B (*p* < 0.05). There was no significant difference in terms of surgical duration between the two groups (*p* > 0.05), as shown in Table 2.

**Table 2:** Perioperative related indicators.

Group	Number of cases	Dosage of propofol (mg)	Surgical duration (min)	Anesthesia recovery time (min)
A	50	77.0 ± 10.2	23.7 ± 5.7	3.6 ± 1.3
B	50	80.3 ± 10.1	22.6 ± 6.3	4.1 ± 1.1
P		0.051	0.329	0.040

The intraoperative propofol dosage, operation time, and postoperative recovery time were compared between the two groups. All data are presented as mean ± SD. A significance level of *p* < 0.05 was considered to indicate a statistically significant difference.

### 3.3 Hemodynamics

The two groups exhibited statistically significant differences in mean arterial pressure (MAP) at various time points, as well as in terms of time and interaction effects (F _group_ = 18.315, *p* < 0.01; F _time_ = 5.019, *p* < 0.05; F _group × time_ = 7.412, *p* < 0.05). Prior to anesthesia administration, there was no significant disparity observed in MAP between the two groups (*p* > 0.05). Group A demonstrated higher MAP values than group B at T1, T2, T3, and T4 (p < 0.05). The MAP at T2, T3, and T4 did not show any statistically significant difference in group A compared to the T0 (*p* > 0.05). However, significant differences were observed at T2, T3, and T4 in group B (*p* < 0.05). No notable distinctions were found between the two groups regarding heart rate and blood oxygen saturation across different time points. as shown in Table 3.

**Table 3:** Vital signs recorded at different time points.

Vital signs	Group	Number of cases	T0	T1	T2	T3	T4
MAP	A	50	89.8 ± 10.34	105.1 ± 10.4^a^	93.6 ± 7.4	93.7 ± 8.0	94.1 ± 6.6
(mmHg)	B	50	91.6 ± 8.9	80.7 ± 3.4^b^	74.3 ± 10.6^ab^	78.3 ± 11.5^ab^	77.7 ± 10.6^ab^
HR	A	50	75.6 ± 10.4	91.9 ± 11.1^a^	82.9 ± 13.2	79.7 ± 12.1	79.9 ± 11.4
(bpm)	B	50	75.6 ± 4.4	86.4 ± 13.0	75.0 ± 9.2	77.2 ± 8.2	77.4 ± 8.0
SpO2	A	50	96.6 ± 0.7	95.6 ± 0.7	96.8 ± 0.5	97.9 ± 0.7	97.7 ± 0.5
(%)	B	50	97.3 ± 0.6	96.9 ± 0.5	96.8 ± 0.3	97.1 ± 0.2	97.0 ± 0.3

MAP, mean arterial pressure, HR, heart rate, SpO_2_, blood oxygen saturation. Data are presented as mean ± SD. Compared with T0, ^a^*p* < 0.05; Compared with Group A, ^b^*p* < 0.05.

## 4 Discussion

Endotracheal interventional therapy under a bronchoscope is safe, effective, and minimally invasive. It has increasingly prominent advantages for treating airway tumors. For patients with definite malignant intratracheal tumors, intratumoral injection of chemotherapy drugs combined with cryotherapy and hyperthermia can play a synergistic role. In previous studies, our team confirmed that drug injection under a bronchoscope can immediately and effectively remove the airway tumor and prolong the time of airway restenosis. However, because bronchoscopy should go deep into the human body, it is an invasive examination that can cause significant discomfort, not only cough but also dyspnea and other serious complications (5). It also leads to operational difficulties and risks to the medical staff (6).

Laryngeal mask airway anesthesia can ensure the airway patency of patients. Hence, they can complete the surgery comfortably. The anesthesia operation stimulation is minimal (7), particularly after applying lidocaine cream. Moreover, the insertion and removal of a laryngeal mask has minimal effects on circulation. General anesthesia can relax the pharyngeal muscles and shorten the surgical duration. At present, opioids are commonly used. However, in the process of anesthesia induction, the blood pressure of patients with hypotension and low HR decreases significantly. Further, vasoactive drugs are required to maintain blood pressure within the normal range.

The sedative and analgesic effects of ketamine have been utilized in clinical anesthesia for over 50 years; however, its prominent side effects, including elevated blood pressure or intracranial pressure, increased airway secretions and heart rate, as well as dizziness and vomiting, have led to its gradual replacement by alternative anesthetic drugs (8). Esketamine is the dextrorotatory enantiomer of ketamine, and its pharmacological properties closely resemble those of ketamine. Both compounds exert anesthetic, amnesic, analgesic, anti-hyperalgesic, and antidepressant effects by inhibiting N-methyl-D-aspartate (NMDA) receptors. Studies have demonstrated that esketamine exhibits approximately three times higher affinity for NMDA receptors compared to ketamine, resulting in a potential 2–3 fold increase in its anesthetic potency (9). The use of esketamine in pediatric short surgery offers several advantages, including the preservation of respiratory muscle tension and protective airway reflexes. Its potent sedative effect effectively caters to the requirements of pediatric surgical procedures. Additionally, its rapid onset and metabolism make it highly suitable for anesthesia in pediatric short surgeries (10, 11). The utilization of esketamine in adult surgery can effectively maintain hemodynamic stability. In comparison to the induction of anesthesia using propofol and sufentanil in elderly surgical patients, the combination of propofol and esketamine exhibits superior hemodynamic stability, while also enhancing surgical stress and inflammation management, as well as reducing anesthesia recovery time. Furthermore, there is no significant difference in the incidence of adverse reactions between sufentanil alone and its combined use with esketamine (12, 13). Other studies have demonstrated that esketamine can partially mitigate the incidence of postoperative cognitive dysfunction in elderly patients undergoing general anesthesia (14). In addition, certain surgical procedures such as breast disease surgery and laparoscopic hysterectomy can impose significant psychological burden on patients. The administration of esketamine following anesthesia induction can effectively enhance postoperative mood in the short term while also exhibiting commendable analgesic properties (15, 16). In this experiment, the intraoperative hemodynamics of patients in the esketamine group exhibited greater stability and a reduction in postoperative recovery time. However, there was a significant elevation in heart rate and blood pressure observed after laryngeal mask placement in the esketamine group. This can be attributed to the systemic release of catecholamines by esketamine, which inhibits norepinephrine reuptake by peripheral nerves, cardiomyocytes, and other neuronal tissues. Additionally, esketamine suppresses vagus nerve activity while enhancing sympathetic nerve excitability. These factors collectively contribute to an increase in heart rate and blood pressure (17, 18). In addition to elevating blood pressure and heart rate, esketamine can also induce an increase in cerebral blood flow, consequently leading to a rise in intracranial pressure. However, under normal circumstances, controlled ventilation or hyperventilation can mitigate the escalation of intracranial pressure caused by esketamine (19, 20). The selection should be made based on the specific conditions of patients in clinical practice.

Sufentanil, an opioid, takes effect 1.5 min after one injection and lasts approximately 40 min. If the dose exceeds the recommended dose, transient respiratory depression may occur. Combined with propofol, it can increase respiratory and circulatory depression, thereby indicating dose-dependent tolerance (21). The advantages of this controlled trial are as follows: First, the analgesic effect is accurate, and a single injection can meet the operation requirements. Second, the recovery of spontaneous respiration was almost unaffected, and recovery was not delayed. However, the main adverse reactions of sufentanil are hypotension and bradycardia (22). In this study, several disadvantages were observed. That is, the blood pressure of patients had a downward trend from anesthesia induction to the end of the surgery.

In this clinical study, the time from anesthesia induction to laryngeal mask insertion to the beginning of surgery was extremely short. Therefore, there was no observation time point between anesthesia induction and the start of interventional surgery.

## 5 Conclusion

Both ketamine and sufentanil can be used as anesthesia for injection therapy under an electronic bronchoscope. However, the effects of these two drugs on blood pressure and HR differ. In clinical practice, the appropriate compatible drug can be selected based on the patient’s vital signs. Therefore, patients can undergo a smoother and more comfortable surgery.

## Data Availability

The raw data supporting the conclusions of this article will be made available by the authors, without undue reservation.
